# Synthesis of Upconversion β-NaYF_4_:Nd^3+^/Yb^3+^/Er^3+^ Particles with Enhanced Luminescent Intensity through Control of Morphology and Phase

**DOI:** 10.3390/nano5010218

**Published:** 2015-02-24

**Authors:** Yunfei Shang, Shuwei Hao, Jing Liu, Meiling Tan, Ning Wang, Chunhui Yang, Guanying Chen

**Affiliations:** 1 School of Chemical Engineering and Technology, Harbin Institute of Technology, Harbin 150001, China; E-Mails: syf19943@sina.com (Y.S.); jing43210@163.com (J.L.); tanmeiling828@163.com (M.T.); wangning9004@sina.com (N.W.); yangchh@hit.edu.cn (C.Y.); 2 Harbin Huigong Technology Co. Ltd, Harbin 150001, China; 3 Institute for Lasers, Photonics and Biophotonics, University at Buffalo, The State University of New York, Buffalo, NY 14260, USA

**Keywords:** NaYF_4_ microcrystals, Nd^3+^ sensitizer, morphology control, upconversion (UC)

## Abstract

Hexagonal NaYF_4_:Nd^3+^/Yb^3+^/Er^3+^ microcrystals and nanocrystals with well-defined morphologies and sizes have been synthesized via a hydrothermal route. The rational control of initial reaction conditions can not only result in upconversion (UC) micro and nanocrystals with varying morphologies, but also can produce enhanced and tailored upconversion emissions from the Yb^3+^/Er^3+^ ion pairs sensitized by the Nd^3+^ ions. The increase of reaction time converts the phase of NaYF_4_:Nd^3+^/Yb^3+^/Er^3+^ particles from the cubic to the hexagonal structure. The added amount of oleic acid plays a critical role in the shape evolution of the final products due to their preferential attachment to some crystal planes. The adjustment of the molar ratio of F^−^/Ln^3+^ can range the morphologies of the β-NaYF_4_:Nd^3+^/Yb^3+^/Er^3+^ microcrystals from spheres to nanorods. When excited by 808 nm infrared laser, β-NaYF_4_:Nd^3+^/Yb^3+^/Er^3+^ microplates exhibit a much stronger UC emission intensity than particles with other morphologies. This phase- and morphology-dependent UC emission holds promise for applications in photonic devices and biological studies.

## 1. Introduction

Lanthanide doped upconversion (UC) materials have attracted increasing interest due to their ability to convert low-energy excitations into high-energy emissions at shorter wavelength [1,2,3]. This unique property makes UC materials suitable for a wide range of applications, such as biological sensing [4,5], *in vivo* imaging [6,7], drug delivery [8,9], and photodynamic therapy [10,11]. Particularly, lanthanide doped fluoride materials revive these applications owing to their higher UC efficiency and excellent physicochemical stabilities [12]. Among them, β-NaYF_4_ is considered to be one of the most efficient host lattices with low phonon energies (<350 cm^−1^) to minimize energy losses at the intermediate states of lanthanide ions [7]. Moreover, Yb^3+^ ions were used as an efficient sensitizer to enhance UC emissions by taking advantage of its high absorption cross-section and efficient energy transferring to the activators of Er^3+^, Ho^3+^, or Tm^3+^. However, the absorption ability of Yb^3+^ sensitizers limited the excitation of most current UC materials to be performed at ~980 nm [13,14]. This elicits heating problems, which cause possible damage to cells and tissues due to strong water absorption at the excitation wavelength.

Recently, extensive efforts have been devoted to development of UC materials that can be excited at better excitation wavelengths but cause minimized adverse effects on the biological cells and tissues. Doping of Nd^3+^ ion as a sensitizer into the current UC systems would be appealing towards this purpose, as Nd^3+^ can be excited by the well-established commercial 808 nm excitation source, the wavelength of which is negligibly absorbed by water molecules. Indeed, the absorption coefficient of water at ~808 nm is around 0.02 cm^−1^, more than 24 times lower than the value of 0.482 cm^−1^ at ~980 nm. The Nd^3+^ sensitizer displays high absorption cross-section (~1.2 × 10^−19^ cm^2^) at 808 nm, which is one order of magnitude higher than that of Yb^3+^ sensitizer (~1.2 × 10^−20^ cm^2^) [15,16]. Moreover, efficient energy transfers have been established from Nd^3+^ to Yb^3+^ and then from Yb^3+^ to the acceptor (Er^3+^, Ho^3+^ or Tm^3+^) [17]. This enables the production of UC materials that can be excited at ~808 nm, and thus draws attention to the need for developing Nd^3+^-sensitized UC materials for biological applications. Yan *et al.* introduced the Nd^3+^ sensitizer into NaGdF_4_ nanoparticles and showed that it minimized the overheating effect in *in vivo* bioimaging compared with Yb^3+^-sensitized nanoparticles [18]. Liu and colleagues presented a new type of core-shell UC nanoparticles based on doping high concentrations of Nd^3+^ ions in the shell structure to enhance the UC emission of activators [13]. Utilizing Nd^3+^-sensitized UC nanoparticles to remove the constraints in conjunction with conventional UC nanoparticles is an important technology for biological studies; this conclusion has also been independently verified in core-shell UC nanoparticles by Han *et al.* and Wang’s group [19,20]. Strategies to prepare Nd^3+^-sensitized particles and to improve and tailor upconverted luminescence from them are in demand in order to produce impetus for their biomedical applications [21,22]. Thus far, very few studies reported on size- and morphology-controlled synthesis of UC Nd^3+^-sensitized particles with tailored luminescence when excited at ~808 nm.

The particle morphologies have been found to play a significant role on the luminescent properties of lanthanide-doped UC materials [23,24,25]. The β-NaLuF_4_:Yb^3+^, Er^3+^ nanomaterials with tube shapes display higher luminescent intensity than those of other morphologies [26]. Lin *et al.* [26] reveal that the luminescent intensity was increased to 6 times in microtubes’ structure, compared with that of limb-like shape. In our previous studies, the microrods exhibit a better photoluminescence signal than other types that resulted in β-NaYF_4_ micro- and nanocrystals [27]. Until now, no attempts have been made to prepare a range of Nd^3+^-sensitized β-NaYF_4_ particles with tuned morphologies and enhanced UC luminescence. Herein, we report on the controlled synthesis of monodisperse Nd^3+^-sensitized β-NaYF_4_ microcrystals and nanocrystals with uniform size and tunable shapes under a hydrothermal condition. We show that variations of the oleic acid ligand and the concentration ratio of F^−^/Ln^3+^ play a synergistic role in defining the morphologies of resulting particles. The crystal growth mechanisms of β-NaYF_4_:10% Nd^3+^, 10% Yb^3+^, 2% Er^3+^ particles with sphere-like shape, octadecahedral shape, and hexagonal shape with protruding centers, were disclosed by collecting samples at pre-set time intervals. Moreover, spectroscopic investigations revealed that upconverted luminescence intensity is dependent on the resulting particle morphology, size, and crystallinity, manifesting the highest intensity from NaYF_4_:10% Nd^3+^, 10% Yb^3+^, 2% Er^3+^ microplates.

## 2. Results and Discussion

### 2.1. Effects of Reaction Time

**Figure 1:**
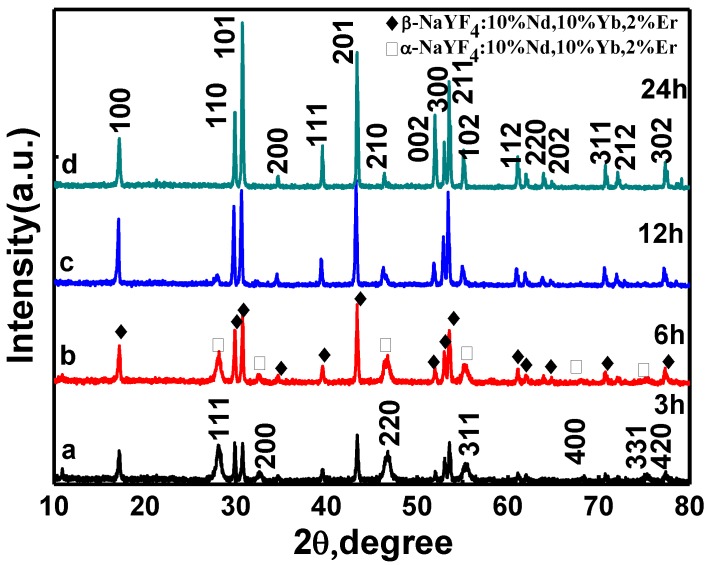
X-ray diffraction (XRD) patterns of NaYF_4_: 10% Nd^3+^,10% Yb^3+^, 2% Er^3+^ microcrystals synthesized at different hydrothermal times: (**a**) 3 h; (**b**) 6 h; (**c**) 12 h; and (**d**) 24 h (180 °C F^−^/Ln^3+^ = 5:1 OA/Ln^3+^ = 40:1).

**Figure 2:**
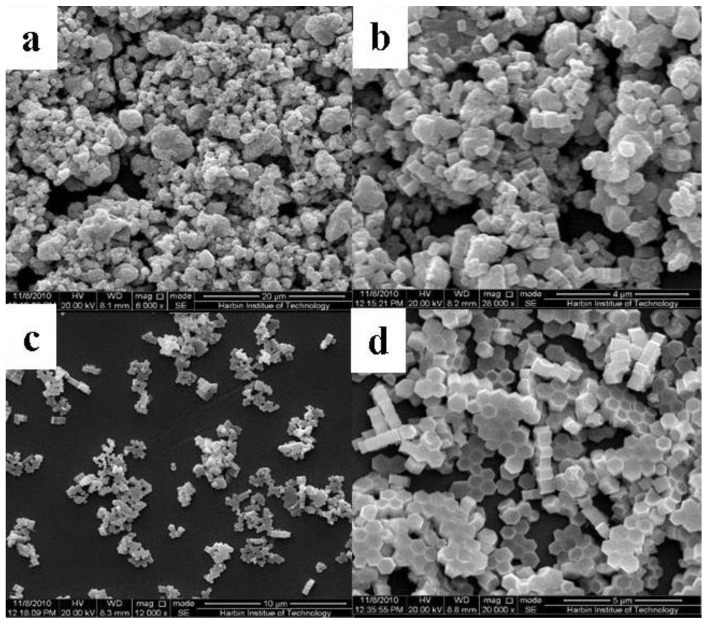
Typical field emission scanning electron microscopy (FESEM) images for the resulting NaYF_4_:10% Nd^3+^, 10% Yb^3+^, 2% Er^3+^ microcrystals prepared with different hydrothermal times: (**a**) 3 h; (**b**) 6 h; (**c**) 12 h; and (**d**) 24 h (Other synthetic parameters were kept identical, 180 °C, F^−^/Ln^3+^ = 5:1, OA/Ln^3+^ = 40:1).

Because the crystal structure of NaYF_4_ exhibits cubic (α-) and hexagonal (β-) polymorphic forms, reaction times were changed to probe the phase transformation process. Figure 1 and Figure 2 show the X-ray diffraction (XRD) patterns and field emission scanning electron microscopy (FESEM) images of the resulting NaYF_4_:10% Nd^3+^, 10% Yb^3+^, 2% Er^3+^ samples, respectively. Figure 1 reveals that the phase transformation process (α→β) occurred gradually with an extended reaction time. The sample obtained with a shorter reaction time (*t* = 3 h) shows a mixture of the α-phase (JCPDS No. 06-0342) and the β-phase (JCPDS No. 16-0334). Judging from the full width of the XRD peaks in Figure 1a, the size of α-phase NaYF_4_ is very small, which agrees well with the SEM result of Figure 2a. As one can see from Figure 2a, the sample includes the aggregates formed by a large quantity of spherical nanoparticles. As the reaction time was extended to 6 h and 12 h, the peak intensity of α-NaYF_4_ became weak (Figure 1b, 1c). In contrast, the peak intensities corresponding to the β-phase NaYF_4_ became stronger. In addition, from the images shown in Figure 2b, 2c, it is clearly seen that hexagonal NaYF_4_ microprisms are obtained, which become more regular and smoother with a longer reaction time. This indicated that the samples transformed gradually from α to β phase as the reaction time was prolonged. When the time was extended to 24 h, only pure β-phase NaYF_4_ existed. Moreover, the intensities of the diffraction peaks in Figure 1d dramatically increased with respect to those samples of short times, implying that the crystallinity of the sample increases with the increase of reaction time. There is a large quantity of microprisms with uniform, smooth, and flat surfaces shown in Figure 2d, which agrees well with the corresponding XRD result of Figure 1d.

The above analysis indicates that the hydrothermal reaction time has significant effects on the size, shape, and phase structure of final products. A long hydrothermal time benefits crystallization and the growth of bigger size microcrystals. Moreover, this result suggests that the β-phase NaYF_4_ crystal particles possess higher thermal energy than the α-phase NaYF_4_; a long hydrothermal time is needed to accumulate adequate energy to complete the phase transformation process.

### 2.2. Effect of the Ratio of OA/Ln^3+^

**Figure 3:**
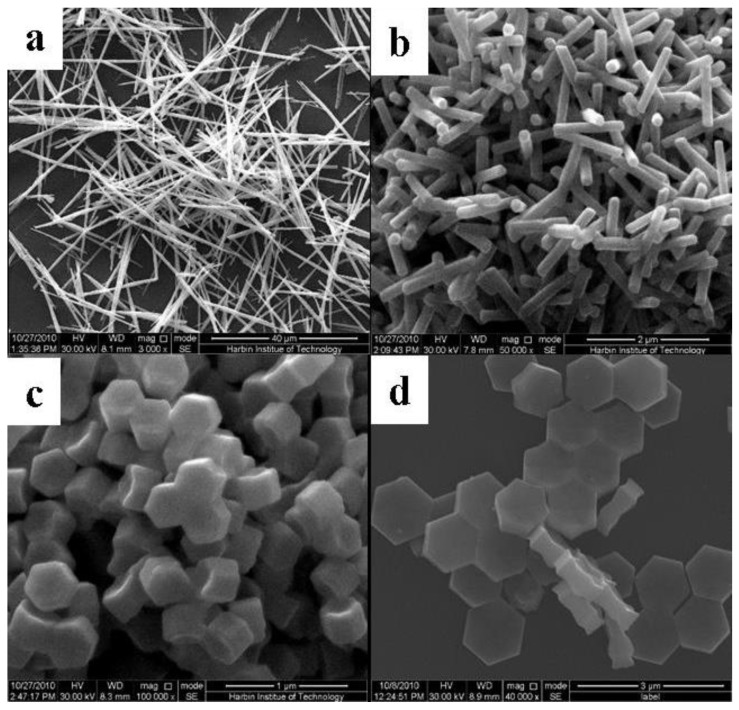
Typical FESEM images for the as-prepared NaYF_4_: 10% Nd^3+^,10% Yb^3+^, 2% Er^3+^ microcrystals at different molar ratios of OA/Ln^3+^: (**a**) 6:1; (**b**) 20:1; (**c**) 30:1; and (**d**) 40:1 (Other synthetic parameters were kept identical, 180 °C, 24 h, F^−^/Ln^3+^ = 5:1).

**Figure 4:**
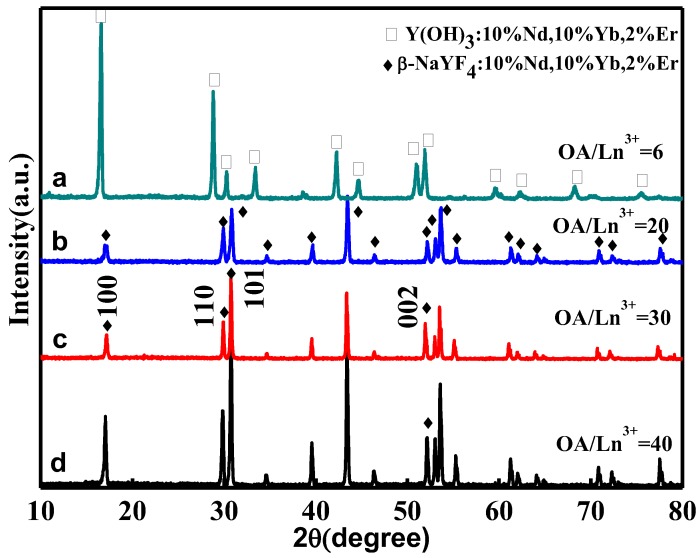
XRD patterns of NaYF_4_:10% Nd^3+^, 10% Yb^3+^, 2% Er^3+^ microcrystals synthesized at different molar ratios of OA/Ln^3+^: (**a**) 6:1; (**b**) 20:1; (**c**) 3:1; and (**d**) 40:1 (Other synthetic parameters were kept identical, 180 °C, 24 h, F^−^/Ln^3+^ = 5:1).

Because OA can rationally control and modify the shape of NaYF_4_ crystals, we probed the role of variation of the molar ratio of OA/Ln^3+^ to impact the resulted morphology of NaYF_4_:10% Nd^3+^, 10% Yb^3+^, 2% Er^3+^ microcrystals in our synthesis system. Figure 3a shows the SEM images of the sample synthesized with 6:1 of OA/Ln^3+^. The image indicates that the resulting products are uniform nanolines. The nanoline can be identified as pure Y(OH)_3_:10% Nd^3+^, 10% Yb^3+^, 2% Er^3+^ by XRD (Figure 4a). It might be explained by the fact that the reaction system was alkaline when it contained little OA. The rare earth ions can thus easily combine with hydroxyl to generate Y(OH)_3_:10% Nd^3+^, 10% Yb^3+^, 2% Er^3+^ precipitation. With the ratio of OA/Ln^3+^ exceeding 20:1, pure β-phase NaYF_4_ was successfully produced, as shown in Figure 4b. Figure 3b shows the SEM images of the sample synthesized with 20:1 of OA/Ln^3+^. The image reveals that the sample contains uniform nanorods with a length of 1.2 µm and an average lateral diameter of 0.245 µm. As the ratio of OA/Ln^3+^ was increased to 30:1, hexagonal microprisms with a length of 0.25 µm and an average lateral diameter of 0.25 µm were obtained. When the ratio of OA/Ln^3+^ exceeded 40:1, uniform hexagonal microprisms with a length of 0.2 µm and the lateral diameter of 1 µm were produced. Hence the aspect ratio L/D (length/diameter) increases gradually with the molar ratio of OA/Ln^3+^. This indicates that the OA plays an important role in the morphology evolution from nanorods to hexagonal microplates. The involved underlined growing mechanism will be discussed in Section 2.4.

### 2.3. Effect of the Ratio of F^−^/Ln^3+^

**Figure 5:**
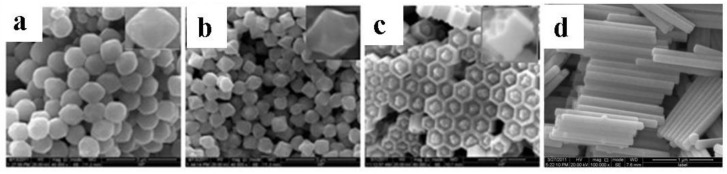

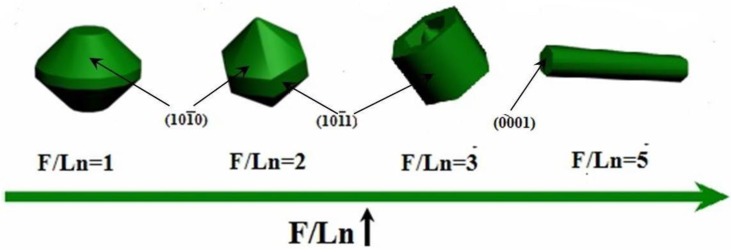
Typical FESEM images for the as-prepared NaYF_4_: 10% Nd^3+^,10% Yb^3+^, 2% Er^3+^ microcrystals at different molar ratios of F^−^/Ln^3+^: (**a**) 1:1; (**b**) 2:1; (**c**) 3:1; and (**d**) 5:1 (Other synthetic parameters were kept identical, 180 °C, 24 h, OA/Ln^3+^ = 20:1).

**Figure 6:**
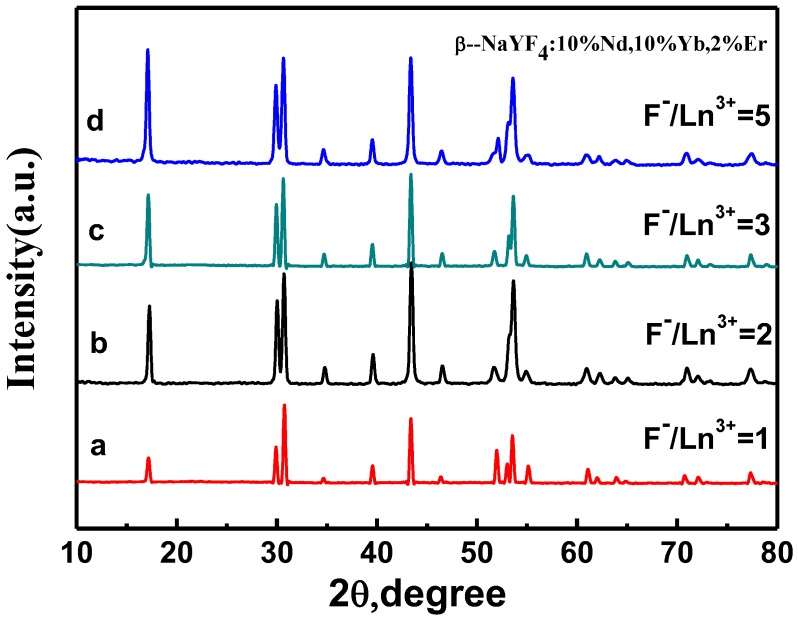
XRD patterns of NaYF_4_:10% Nd^3+^,10% Yb^3+^, 2% Er^3+^ microcrystals synthesized at different molar ratios of F^−^/Ln^3+^: (**a**) 1:1; (**b**) 2:1; (**c**) 3:1; and (**d**) 5:1 (Other synthetic parameters were kept identical, 180 °C, 24 h, OA/Ln^3+^ = 20:1).

To investigate the effect of the F^−^/Ln^3+^ (F^−^/Ln^3+^ ≤ 5) ratio on the morphology, size, and structure of the as-prepared NaYF_4_:10% Nd^3+^, 10% Yb^3+^, 2% Er^3+^ particles, the ratio was taken as 1:1, 2:1, 3:1, and 5:1, respectively. As shown in Figure 5, with a gradual increase of the molar ratio of F^−^/Ln^3+^, a significant change takes place in the morphology and size of NaYF_4_:10% Nd^3+^, 10% Yb^3+^, 2% Er^3+^ microcrystals. When the F^−^/Ln^3+^ ratio is fixed at 1:1, the as-prepared microcrystals are spherical with coarse surfaces, implying low crystallization (Figure 5a). When the molar ratio of F^−^/Ln^3+^ increased to 2:1 and 3:1, hexagonal microprisms with a protruding center and distortional tubular structure—with the end face of the central convex and concave shape between the center and the edge—were obtained. In addition, with the increase of F^−^ concentration, the crystallization is also improved. As the molar ratio of F^−^/Ln^3+^ exceeded 5:1, uniform nanorods with high crystallization were generated. It can be seen that, with the increase of the molar ratio of F^−^/Ln^3+^, each surface gradually became smooth as along with the higher crystallization, which has been proved by XRD patterns (Figure 6). All the peaks of each sample are characteristic of a pure β-phase of NaYF_4_; however, the relative intensities are different from each other, implying a different preferential orientation growth.

### 2.4. The Mechanism of NaYF_4_ Crystal Growth

The hydrothermal growth process of NaYF_4_ microcrystals includes the nucleation and the growth steps. In the process of nucleation, the OA anions could serve as chelating agent in reaction solution and formed complexes with Y^3+^ ions due to the strong coordination interaction. The chelating ability of the Y^3+^-OA complex would be weakened under high hydrothermal temperature and pressure, and thus gradually release the Y^3+^ ions to react with Na^+^ and F^−^ ions to generate the NaYF_4_ nuclei. During the process of growth, OA as stabilizing agents can attach to the surfaces of NaYF_4_ microcrystal with the alkyl chains left outside, which controls the growth of microcrystals and provides steric forces to prevent their aggregations.

To test our supposition, we have carried out two parts of the synthetic experiment.

**Scheme 1:**
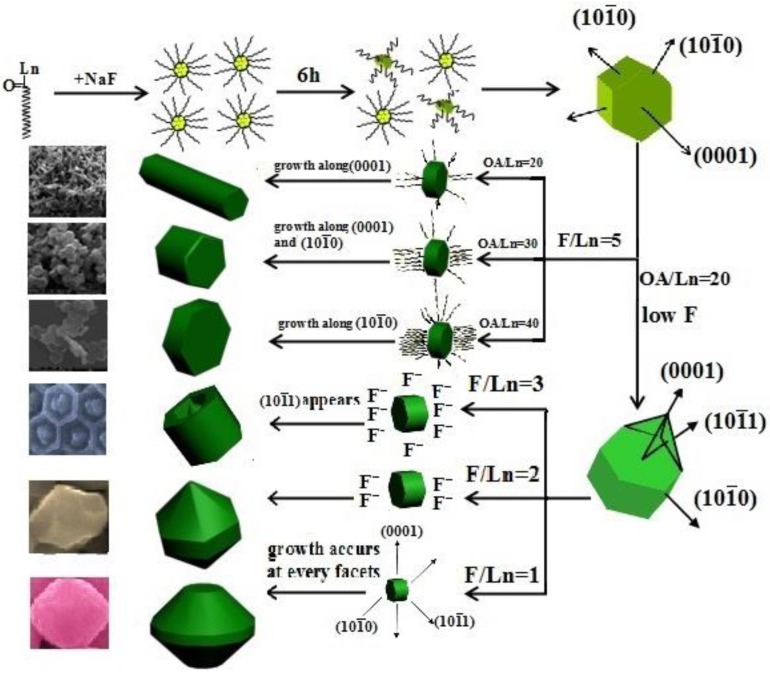
Schematic illustration of the possible formation mechanism of NaYF_4_ with various morphologies under different OA/Ln^3+^ and F^−^/Ln^3+^ molar ratio conditions.

**Part 1:** the molar ratio of F^−^/Ln^3+^ was fixed at 5, and then the molar ratio of OA/Ln^3+^ was changed from 20:1 to 40:1. The hexagonal NaYF_4_ seed generally includes (0001) and (1010) facets, as shown in Scheme 1. It is noted that the facets with smaller surface area will have a higher surface energy and a faster growth rate, thus impacting the shapes of the final products. As for the hexagonal NaYF_4_, the area of (1010) exceeds that of (0001). This indicates that (0001) facets have higher surface energy, and thus a higher growth velocity, *i.e.*, *v*(0001) *>> v*(1010). When the amount of oleic acid (OA) is relatively low (OA/Ln^3+^ < 20:1), OA molecules preferentially adsorb onto the (0001) facets, thus producing uniform microrods (Figure 3b). The short microrods with an average diameter of 0.25 µm and a height of 0.245 µm can be achieved when the OA /Ln^3+^ is increased to 30:1 (Figure 3c). When more OA molecules are introduced, the ratio of *v*(1010)/*v*(0001) will be increased, as the number of OA molecules on the (0001) facet will saturate but will get larger on the (1010) facet with larger surface area. When *v*(0001) *= v*(1010), the resulting particles will simultaneously grow along the (0001) and (1010) directions to shorter microrods. When this ratio was decreased below 1 as more OA was introduced, particles with microplates formed.

**Part 2:** the molar ratio of OA/Ln^3+^ was fixed at 20, while the molar ratio of F^−^/Ln^3+^ varied from 1:1 to 5:1. We found that this condition provided an ideal system to induce OA and F^−^ preferential adsorption onto (0001) facets to control the growth rates of different facets of NaYF_4_ microcrystals. As can be seen in Figure 5, the hexagonal NaYF_4_ seed crystals have another type of surface (1010) in the case of F^−^/Ln^3+^ < 5. The nucleation and growth rate was thus controlled through the competition between F^−^ and OA towards (0001), (1010), and (1011) facets of NaYF_4_ microcrystals. The XRD patterns of the products obtained at various ratios of F^−^/Ln^3+^ (1:1, 2:1, 3:1, and 5:1) are shown in Figure 6a–d, respectively. As can be seen, all the peaks of each sample are characteristic of a pure β-phase of NaYF_4_, but the relative intensities are different from each other, suggesting different preferential orientation growth. When the amount of OA(OA/Ln^3+^ = 20) is not enough to hinder the growth of (0001) facets, the differences in the molar ratios of F^−^/Ln^3+^ have a significant effect on the growth of NaYF_4_ microcrystals. With a low molar ratio of F^−^/Ln^3+^(1:1), all of the F^−^ ions are used in nucleation. Accordingly, the growth of every facet is similar, thus resulting in sphere-like microcrystals (Figure 5a). When the molar ratio of F^−^/Ln^3+^ is increased to 2:1, the remainder of the F^−^ ions attacks the high-energy (0001) facet. Simultaneously, owing to the low free energies of the circumferential edges of seeds, the redundant F^−^ ions preferentially bound to the circumferential edges of seeds, which would result in growth along the circumferential edge direction [27,28]. The concave octadecahedral β-NaYF_4_ microcrystals can thus be produced. At F^−^/Ln^3+^ = 3:1, the different crystallographic planes can be recognized in Figure 5c. The presence of conical ends with a raised ridge demonstrates that the (1011) facet appears. Moreover, in comparison with octadecahedral β-NaYF_4_ microcrystals (F^−^/Ln^3+^ = 2:1), the length enhancement further confirms that the growth rate is in the (0001) facet. However, in the condition F^−^/Ln^3+^ = 5:1, uniform β-NaYF_4_ nanorods are produced. This is possibly because of the large amount of F^−^ ions binding strongly to (0001) surfaces to speed up the growth ofβ-NaYF_4_ crystallites.

### 2.5. UC Luminescence Properties

**Figure 7:**
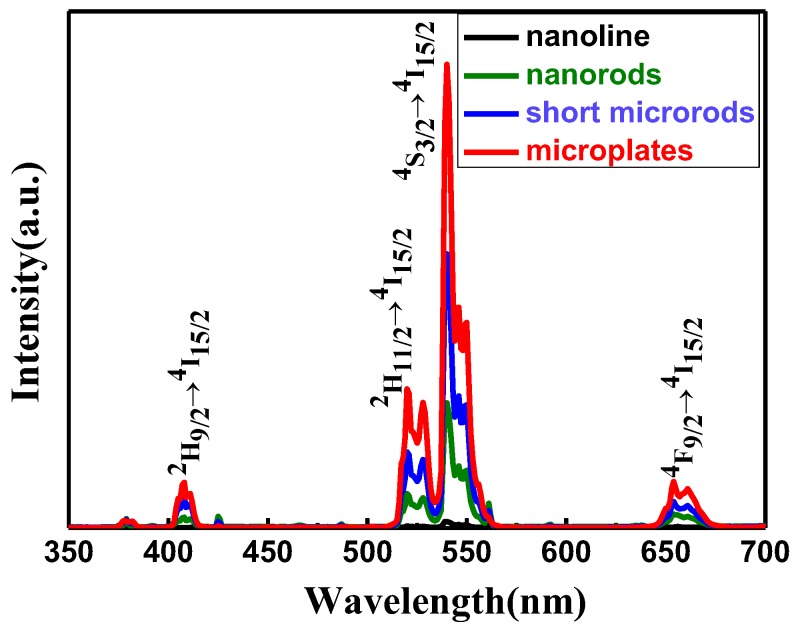
UC Photoluminescence (PL) spectra of NaYF_4_:10% Nd^3+^,10% Yb^3+^, 2% Er^3+^ microcrystals with different molar ratios of OA/Ln^3+^ under diode laser excitation at 808 nm. Excitation power density, ~390 W/cm^2^.

**Figure 8:**
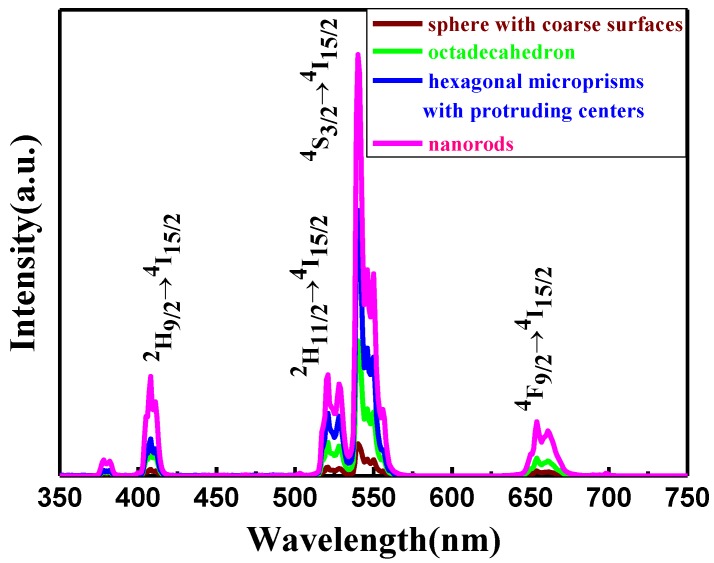
UC Photoluminescence (PL) spectra of NaYF_4_:10% Nd^3+^,10% Yb^3+^, 2% Er^3+^ microcrystals with different molar ratios of F^−^/Ln^3+^ (180 °C, 24 h, OA/Ln^3+^ = 40) under diode laser excitation at 808 nm. Excitation power density, ~390 W/cm^2^.

β-NaYF_4_ is regarded as an efficient host for the UC process due to low phonon energies. Nd^3+^ ions were selected as the sensitizer to investigate the spectral and UC luminescent properties of products with various morphologies. Figure 7 shows the UC PL emission spectra of the resulting β-NaYF_4_:10% Nd^3+^, 10% Yb^3+^, 2% Er^3+^ phosphors prepared at 180 °C and the F^−^/Ln^3+^ ratio = 5:1 when changing the OA/Ln^3+^ ratio from 6:1 to 40:1 upon irradiation of 808 nm wavelength. UC emission peaks of purple, green, and red centered at 410 nm, 520/540 nm, and 655 nm were observed, which can be assigned to the ^2^H_9/2_ → ^4^I_15/2_, ^2^H_11/2_/^4^S_3/2_ → ^4^I_15/2_, and ^2^H_9/2_ → ^4^I_15/2_ transitions of Er^3+^ ions, respectively. It is interesting to note that the shapes of the emission spectra are similar in all four samples, with the only difference being in the relative intensities of the bands. Additionally, the microplate crystals show highest emission intensity in the four samples. This difference in UC luminescence intensity may be attributed to the changes in morphology, crystallinity, and particle size [29,30]. Excluding the Y(OH)_3_ nanoline, the results show that the β-NaYF_4_: 10% Nd^3+^,10% Yb^3+^, 2% Er^3+^ microcrystals with high crystallinity and large size emit stronger UC light owing to the low surface-to-volume ratio. The smaller the surface-to-volume ratio of a microcrystal, the fewer active ions are located on the surface of particles. Therefore, microplates with large size and high crystallinity show a small surface defect, which is efficient to restrain the photon quenching. Moreover, in our case, similar results have also been observed by tuning the molar ratio of F^−^/Ln^3+^ from 1:1 to 5:1 (shown in Figure 8). The strongest emission has been observed for nanorods with relatively large size and high crystallinity.

**Figure 9:**
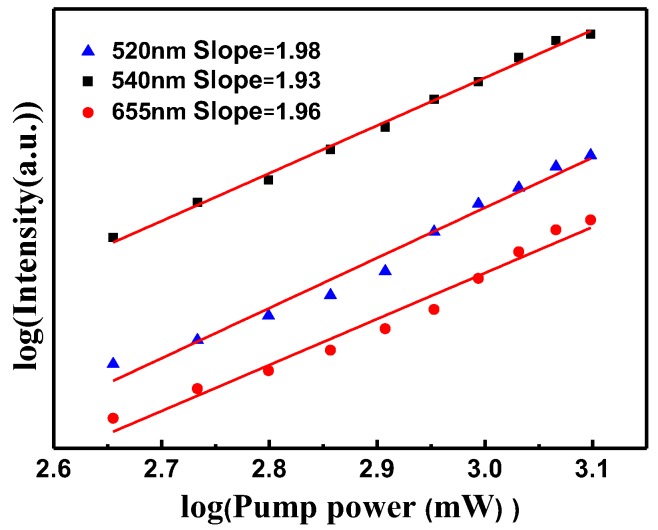
Pump power dependence of the fluorescent bands centered at 520, 540, and 655 nm from NaYF_4_:10% Nd^3+^, 10% Yb^3+^, 2% Er^3+^ on pumping power.

In order to investigate the UC luminescence mechanism, pump power dependences of the luminescence bands centered at 520, 540, and 655 nm were measured and displayed in a double logarithmic plot in Figure 9. For the unsaturated case, the number of photons that are required to populate the upper emitting state can be obtained by the relation *I*_f_ ∝ *P^n^*, where *I*_f_ stands for the fluorescent intensity, *P* is the pump laser power, and *n* is the number of laser photons required. Fitting the data points yielded slope values of 1.98, 1.93, and 1.96 for the ^2^H_11/2_ → ^4^I_15/2_, ^4^S_3/2_ → ^4^I_15/2_, and ^4^F_9/2_→^4^I_15/2_ transitions, respectively. This demonstrates that the generation of UC luminescence through the Nd^3+^-sensitized system involves a two-photon process. In the Nd^3+^, Yb^3+^, and Er^3+^ tri-doped system, the energy transfer from Nd^3+^ to Yb^3+^ and then to Er^3+^ should be taken into account under 808 nm diode laser excitation.

**Figure 10:**
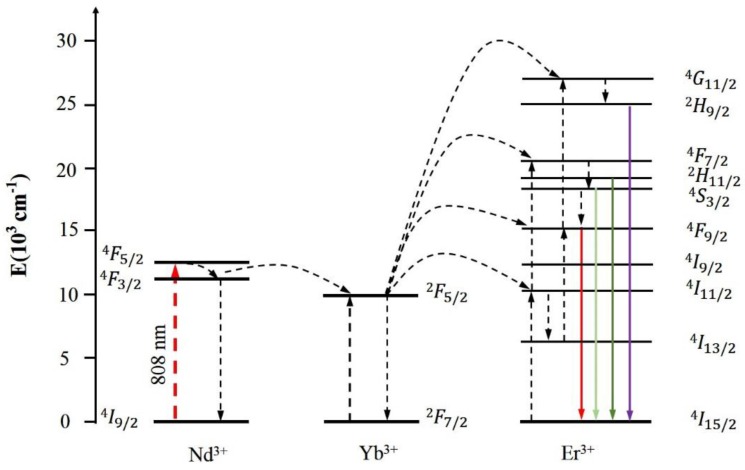
Proposed energy transfer mechanism of Nd^3+^, Yb^3+^, Er^3+^ ions following diode laser excitation of 808 nm. The dashed-dotted, dashed, dotted, and full arrows represent the photon excitation, energy transfer, and emission processes.

The energy transfer mechanism was displayed in Figure 10. The sensitizer Nd^3+^ ions were firstly excited from the ^4^I_9/2_ to the ^4^F_5/2_ state after absorbing the excitation energy at 808 nm, and then relaxed to the ^4^F_3/2_ level through multiphonon processes. The Nd^3+^ ions transfer their absorbed energy to neighboring Yb^3+^ ions and excite them from the ^2^F_7/2_ (Yb^3+^) to the ^2^F_5/2_ (Yb^3+^) state. Subsequently, the Yb^3+^ ion at ^2^F_5/2_ state transfers the received energy to its neighboring Er^3+^ ion, exciting it from ^4^I_15/2_ (Er^3+^) to ^4^I_11/2_ (Er^3+^) stare. Receiving transferred energy from the Yb^3+^ ion can further excite the Er^3+^ ion to the ^4^F_7/2_ (Er^3+^) level. Multiphonon-assisted relaxations from the ^4^F_7/2_ state can then populate the ^2^H_11/2_ (Er^3+^) and ^4^S_3/2_ (Er^3+^) levels, which generate the 520 and 540 nm emissions by radiative decay to the ground state. In addition, the red emission around 655 nm can be acquired through transition from ^4^F_9/2_ (Er^3+^) to ^4^I_15/2_ (Er^3+^). At the same time, a portion of the Er^3+^ ions at ^4^F_9/2_ state can receive energy transfers from Yb^3+^ ions and be promoted to the ^4^G_11/2_ state, from which the ^2^H_9/2_ state can be populated through nonradiative relaxation process. Decay of the excited Er^3+^ ions in ^2^H_9/2_ state to ^4^I_15/2_ state results in the purple emission centered at 410 nm.

## 3. Experimental Section

### 3.1. Preparation

The NaYF_4_ microcrystals were hydrothermally prepared using oleic acid as shape modifier, and NaF, Y(NO_3_)_3_ as precursors at 180 °C under a basic condition. In a typical synthesis of NaYF_4_ nanorods, 1.08 g (27 mmol) of NaOH, 9 mL (24 mmol) of oleic acid (OA) (90 wt.%), and 10 mL (120 mmol) of ethanol were well mixed at room temperature to get a white viscous solution. Then, 4 mL (3.6 mmol) of 0.252g NaF (F^−^/Ln^3+^ = 5:1) solution was added with vigorous stirring until a translucent solution was obtained. Then 6.41 mL (1.2 mmol) of 0.1872 M Y(NO_3_)_3_ was poured into the above solution keeping vigorous stirring. After aging for 1 h, the mixture was transferred to a 50-mL Teflon-lined autoclave, and heated at 180 °C for 24 h. The NaYF_4_ microplates were prepared under similar conditions except that the amount of OA doubled. It is noted that the total molar composition of rare earth metals remains constant. For example, to prepare NaYF_4_ doped with 10% Nd^3+^, 10% Yb^3+^, 2% Er^3+^, 5 mL (0.936 mmol) 0.1872 M Y(NO_3_)_3_, 1 mL (0.12 mmol) 0.12 M Nd(NO_3_)_3_, 1 mL (0.12 mmol) 0.12 M Yb(NO_3_)_3_, and 1 mL (0.024 mmol) of 0.024 M Er(NO_3_)_3_ were mixed together instead of 6.41 mL of 0.1872 M Y(NO_3_)_3_. The obtained microcrystals were washed with ethanol and water to remove the oleic acid and other remnants, and then dried in the air at 60 °C for 12 h.

### 3.2. Characterization

The as-prepared samples were characterized by X-ray powder diffraction (XRD) on a Rigaku D/max-γB diffractometer, which was equipped with a rotating anode and a Cu Kα source (λ = 0.154056 nm). Micrographs of the prepared powders were obtained by using a field emission scanning electron microscope (FESEM, MX2600FE, AIKE Sepp, Oxford, UK). To measure the emitted UC luminescence, the synthesized powders were pressed to form a smooth, flat disk, which was irradiated with a focused 5 W power-controllable 808 nm diode laser (Hi-Tech Optoelectronics Co. Ltd, Beijing, China). The emitted UC luminescence was then collected by a lens-coupled monochromator (Zolix Instruments Co. Ltd, Beijing, China) of 2 nm spectral resolution with an attached photomultiplier tube (Hamamatsu CR131, Hamamatsu Photonics, Hamamatsu, Japan).

## 4. Conclusions

In conclusion, we have presented our systematic synthesis results on hexagonal NaYF_4_:10% Nd^3+^, 10% Yb^3+^, 2% Er^3+^ micro- and nanocrystals with various morphologies and size. Morphologies with nanorods, short microrods, and microplates can be successfully achieved by varying the amount of OA coordination ligand in the initial precursor solution. This is ascribed to the fact that the OA is able to modulate the growth rate of (0001) and (1011) crystallographic facets due to their preferential attachment to them, thus governing the formation of the final morphologies. Moreover, through tuning the molar ratio of F^−^/Ln^3+^, sphere-like, octadecahedral, and hexagonal microprisms with protruding centers NaYF_4_:10% Nd^3+^, 10% Yb^3+^, 2% Er^3+^ can be obtained. This shape evolution is a direct cooperative result between the attachment of F^−^ and OA to the involved crystal facets of (0001), (1011), and (1010). Furthermore, under 808 nm laser excitation and the same measurement conditions, spectroscopic investigations of the resulting particles revealed a morphology-, size-, and crystallinity-dependent upconverted luminescence; the highest luminescence intensity was from NaYF_4_:10% Nd^3+^, 10% Yb^3+^, 2% Er^3+^ microplates. These Nd^3+^-sensitized hexagonal NaYF_4_ nanoparticles with various size and shape have important implications for biophotonic applications with minimized heating effects.

## Supplementary Materials

Supplementary File 1
