# Supplementary material for: Synthesis of Upconversion β-NaYF4:Nd3+/Yb3+/Er3+ Particles with Enhanced Luminescent Intensity through Control of Morphology and Phase
Source: Nanomaterials (Basel). 2015 Feb 24;5(1):218–32. doi: 10.3390/nano5010218 (PMC5312859; doi:10.3390/nano5010218)
Supplement: Supplementary File 1 [file nanomaterials-05-00218-s001.pdf]

Supplementary Information

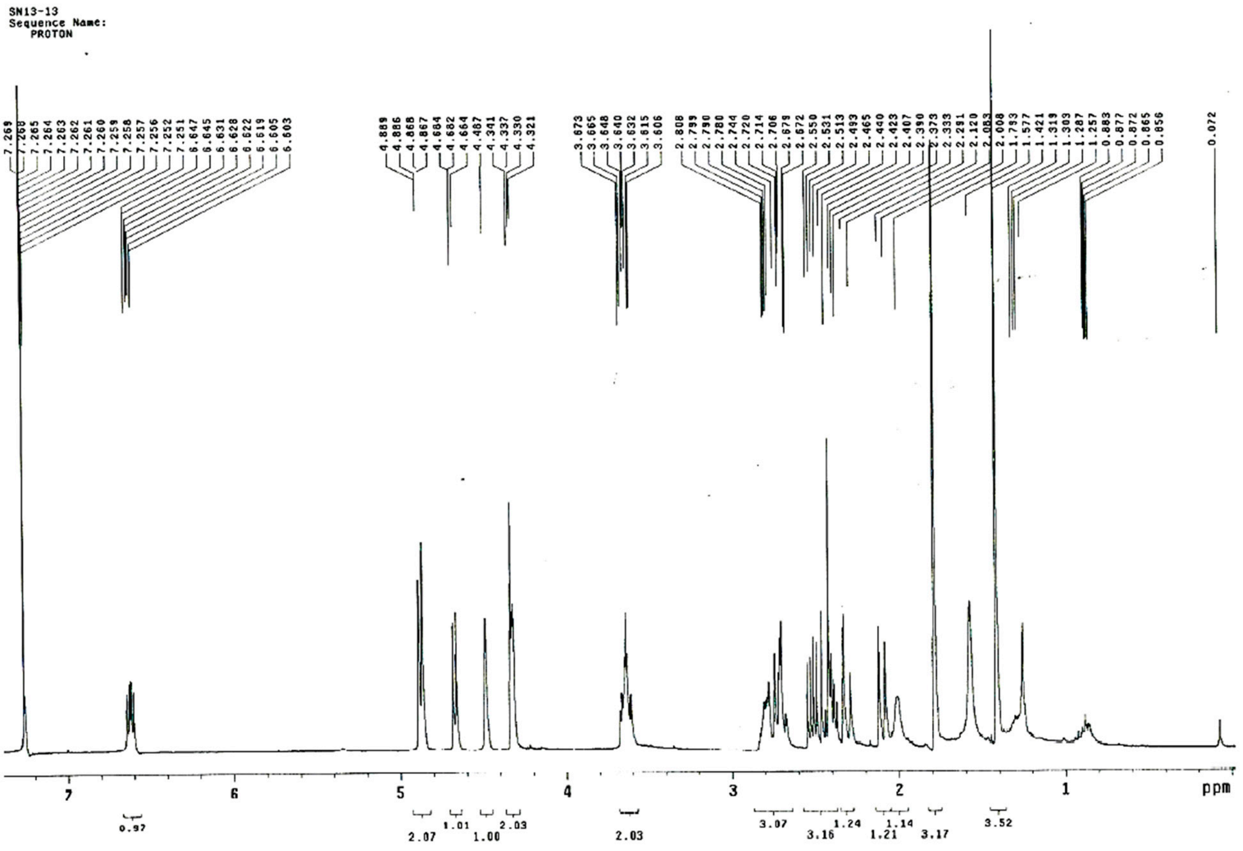

Figure S1. ^{1}H NMR spectrum (400 MHz) of compound **2** in $CDCl_3$.

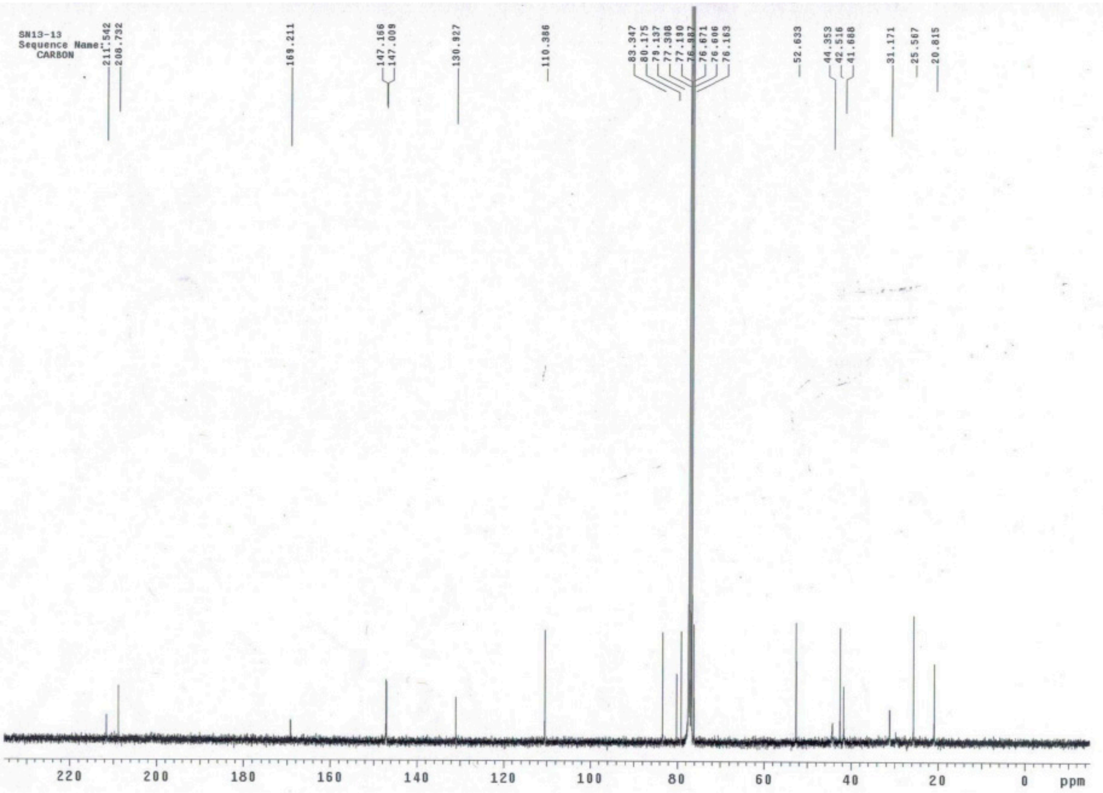

Figure S2. ^{13}C NMR spectrum (100 MHz) of compound **2** in $CDCl_3$.

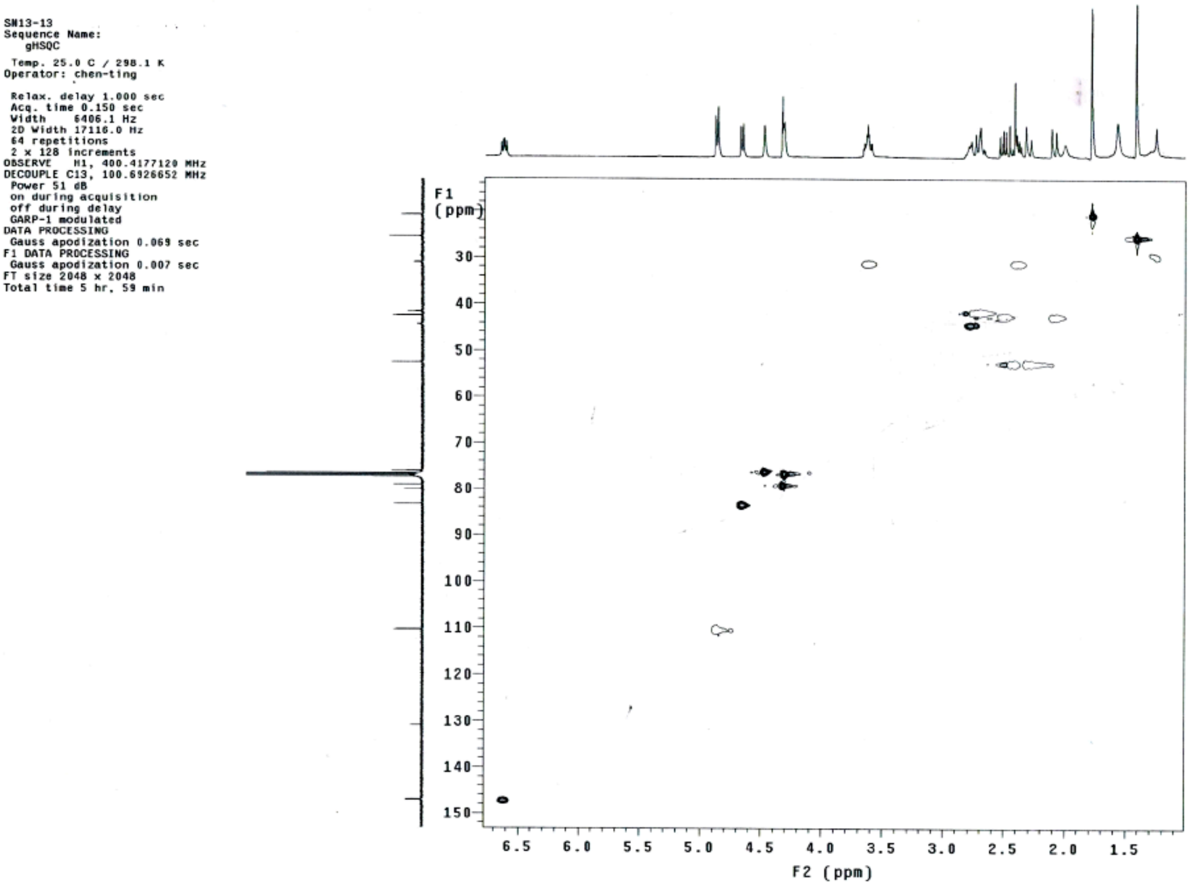

Figure S3. gHSQC spectrum (400 MHz) of compound **2** in $CDCl_3$.

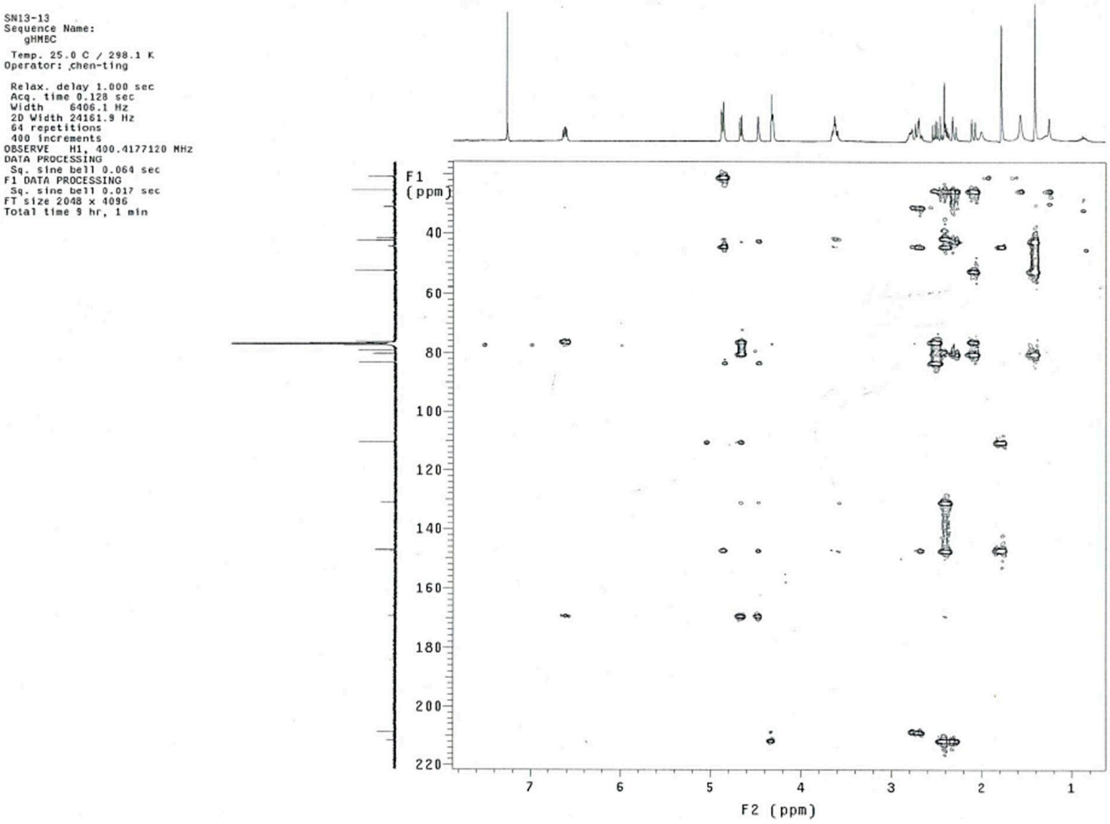

Figure S4. gHMBC spectrum (400 MHz) of compound **2** in $CDCl_3$.

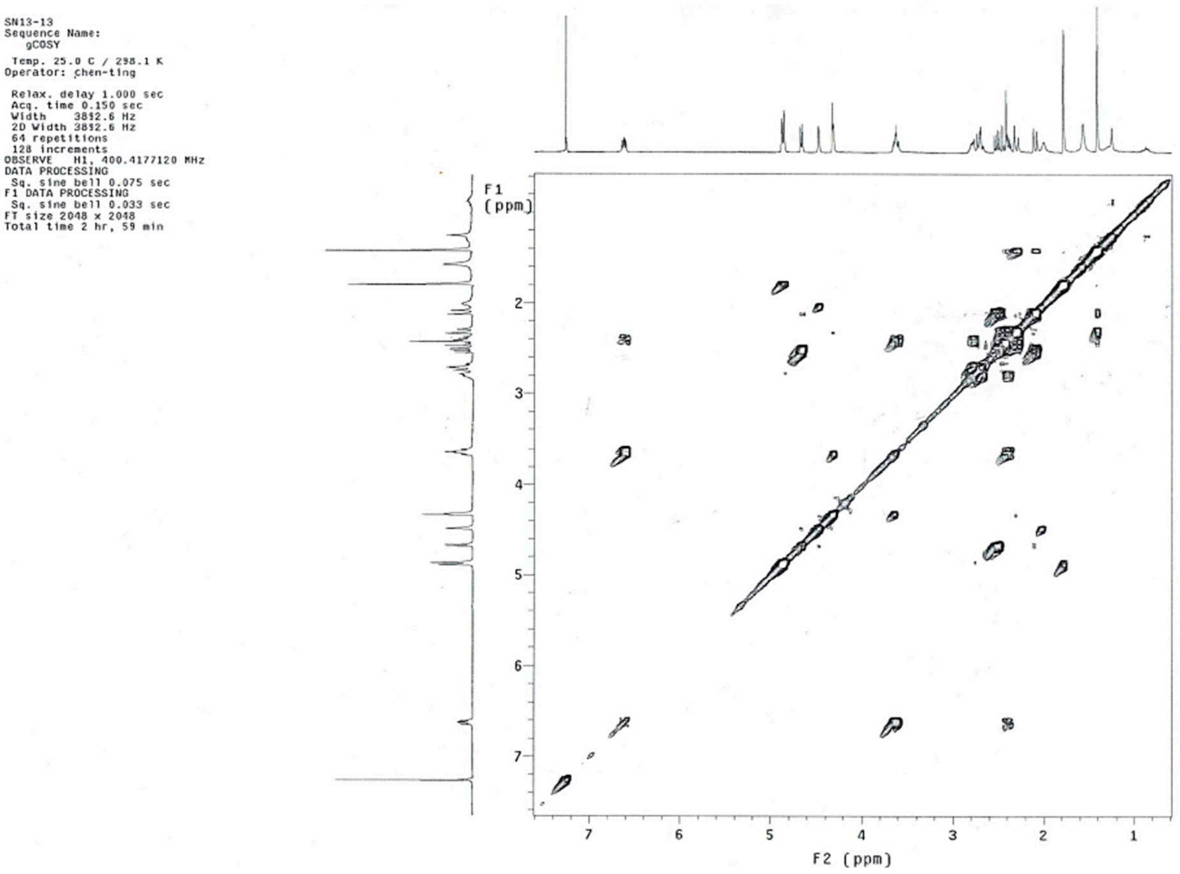

Figure S5. COSY spectrum (400 MHz) of compound **2** in $CDCl_3$.

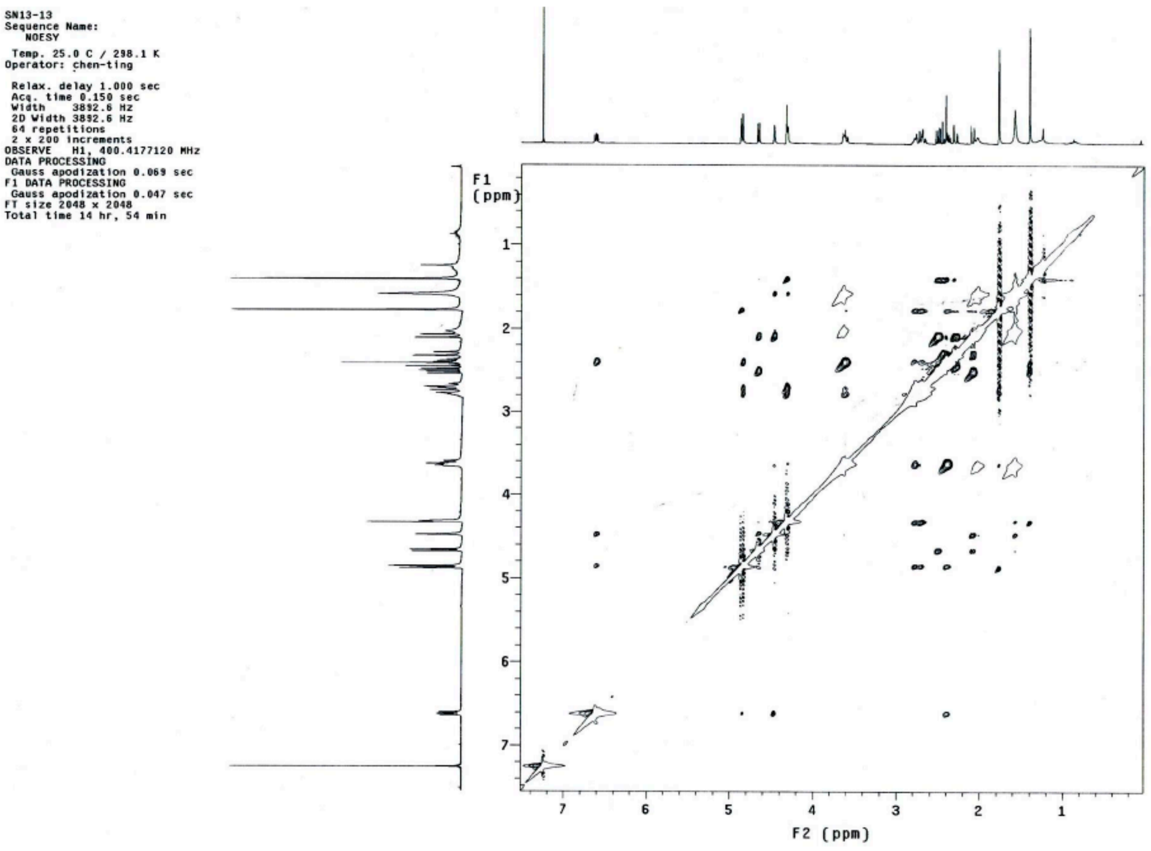

Figure S6. NOESY spectrum (400 MHz) of compound **1** in $CDCl_3$.

Mass Spectrum SmartFormula Report

Analysis Info

Analysis Name	D:\Data\a2\SN1313_000003.d	10/23/2014 3:41:51 PM
Method	broadband first signal	
Sample Name	SN13-13	Instrument: FT-MS solariX
Comment	ESI Positive	

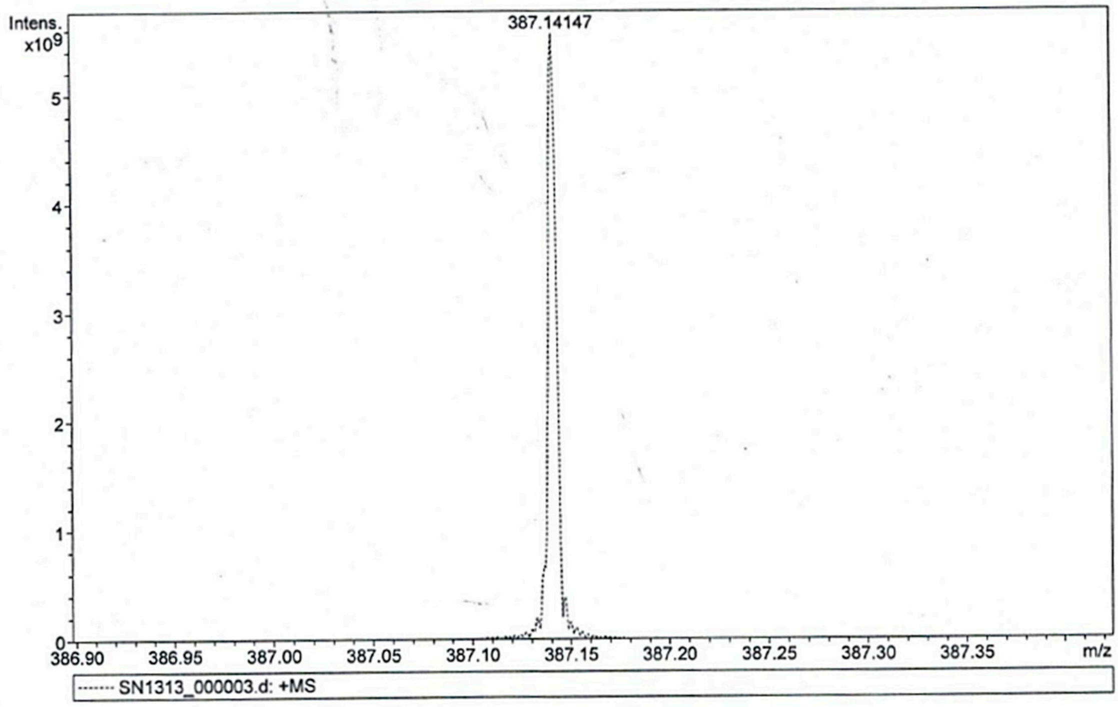

Meas. m/z	#	Formula	Score	m/z	err [mDa]	err [ppm]	mSigma	rdb	e⁻ Conf	N-Rule
387.14147	1	C 19 H 24 Na O 7	100.00	387.14142	-0.05	-0.12	11.2	7.5	even	ok

Figure S7. HRMS of compound **2**.
